# Accurately mapping the location of the binding site for the interaction between hepatitis B virus X protein and cytochrome *c* oxidase III

**DOI:** 10.3892/ijmm.2014.2018

**Published:** 2014-12-02

**Authors:** DAN LI, JIAN DING, ZHIXIN CHEN, YUN CHEN, NA LIN, FENGLIN CHEN, XIAOZHONG WANG

**Affiliations:** 1 Department of Gastroenterology, Union Hospital of Fujian Medical University, Fuzhou, Fujian 350001, P.R. China; 2 Department of Gastroenterology, First Affiliated Hospital of Fujian Medical University, Fuzhou, Fujian 350001, P.R. China

**Keywords:** co-immunoprecipitation, cytochrome *c* oxidase subunit III, hepatitis B virus, hepatitis B virus X protein, mitochondria, yeast two-hybrid system

## Abstract

The hepatitis B virus (HBV) X protein (HBx) plays an important pathogenetic role in hepatocarcinoma tumorigenesis. As HBx does not have the ability to bind to double-stranded DNA (dsDNA), protein-protein interaction is crucial for HBx functions. In a previous study, we screened a novel HBx-interacting protein, the cytochrome *c* oxidase subunit III (COXIII). In the present study, we aimed to accurately map the location of the binding site for the interaction of HBx with COXIII. Two fragments of HBx mutants (X1 aa1-72 and X2 aa1-117) were amplified by polymerase chain reaction (PCR) and separately inserted into the pAS2-1 plasmid. PCR and gene sequencing confirmed the correct insertion of the mutant fragments in the plasmid. The tanscription of the mutant fragments in yeast cells was demonstrated by RT-PCR and western blot analysis confirmed that they were accurately translated into fusion proteins. Hybridization on solid medium and the detection of β-galactosidase (β-gal) activity indicated that the binding site for the interaction between HBx and COXIII was located between aa72 and aa117. Specific interactions between the HBxX2 protein and COXIII were verified by co-immunoprecipitation. To the best of our knowledge, this is the first study showing to demonstrate that aa72-117 in HBx is the key region for binding with COXIII.

## Introduction

Human hepatitis B virus (HBV) infection is strongly associated with the development of hepatocellular carcinoma (HCC) (1). It contains a unique open reading frame, the sequence of which is highly conserved among different mammalian hepadnaviruses and codes for a 16.5-kDa protein known as hepatitis B virus X protein (HBx). The mechanisms through which HBV causes malignant transformation remain unelucidated; however, available evidence supports a pathogenetic role for the product of the HBV X gene, HBx (2). A large number of putative mechanisms through which HBx may contribute to the development of HCC have been investigated. Studies on HBx-responsive elements have indicated that HBx transactivates viral and cellular genes through transcriptional regulatory factors, such as nuclear factor (NF)-κB, activator protein (AP)-1, AP-2 and cAMP response element binding protein (CREB). It also interacts with a group of transcriptional co-activators that includes TATA-binding protein-associated factors (3,4). However, the molecular mechanisms responsible for the effects of HBx on transcription, cellular proliferation and transformation are only partially defined. As HBx does not have the ability to bind to double-stranded DNA (dsDNA), protein-protein interaction is crucial for HBx functions. The interactions of HBx with cellular proteins may trigger a cascade of phosphorylation and dephosphorylation events that lead to a general upregulation of gene expression (5). The identification of cellular proteins that interact with HBx may provide insight into the mechanisms through which HBV exerts its cellular effects. In our previous study, using the *Saccharomyces cerevisiae* two-hybrid system, we found a novel HBx-interacting protein that is homologous to the *Homo sapiens* cytochrome *c* oxidase III (COXIII) (6). In the present study, once again using the yeast two-hybrid system, we took two fragments of the HBV X gene mutants, that are translated as HBx X1 aa1-72 and X2 aa1-117, inserted them separately into plasmids, and screened them to identify the fragment that interacts with COXIII. The results indicated that the binding site for the interaction between HBx and COXIII was located between aa72-117. The data presented herein may form the basis for future studies on HBx interactive elements and may provide insight into the mechanisms through which HBx causes malignant transformation in HCC.

## Materials and methods

### Materials

The *Saccharomyces cerevisiae* AH109 yeast strain was grown in YPD medium (10 g/l yeast extract, 20 g/l peptone and 20 g/l dextrose) or synthetic minimal medium (0.67% yeast nitrogen base, 2% dextrose and appropriate auxotrophic supplements) following transfection. The media were from Clontech Laboratories, Inc. (Mountain View, CA, USA). The yeast strain carries the *LacZ*, *HIS3* and *ADE2* reporter genes under the control of the Gal4 binding site and has deletions in the *trp* and *leu* genes. Y187-pACT2-COXIII (*Saccharomyces cerevisiae* Y187 yeast strain with a *LacZ* reporter gene and deletions in the *trp* and *leu* genes, carrying pACT2 expressing the full-length COXIII gene) was obtained from our institution. The plasmids, pAS2-1 (which includes the binding domain for the detection of the fusion protein), pCL1, pUCm-T, pCMV-HA and pLAM5′-1, were from Clontech Laboratories, Inc.. pcDNA3-X, pAS2-1-X, pCMV-Myc-COXIII, pCMV-HA-X and pUCm-T-X were constructed and maintained at our institution (6). The binding domain and c-Myc monoclonal (Cat. no. 631206) and hemagglutinin (HA) polyclonal (Cat. no. 631207) antibodies were also provided by Clontech Laboratories, Inc.. The mouse anti-human β-actin antibody was from Santa Cruz Biotechnology, Inc. (Dallas, TX, USA). The *Eco*RI, *Xho*I and *Pst*I restriction enzymes, T4 DNA ligase, Taq DNA polymerase, dNTP, the Plasmid DNA purification system and the TransFast transfection reagent kit were from Promega Biosciences LLC (San Luis Obispo, CA, USA). The DNA ladder was from Life Technologies Corp. (Carlsbad, CA, USA). The glass beads were from Sigma-Aldrich Corp. (St. Louis, MO, USA). The DNA gel extraction kit was from Shanghai Sangon Biotech (Shanghai, China). The electrochemiluminescence kit was from Boster Biotech (Wuhan, China).

#### Methods

### Plasmid construction

*Escherichia coli* (*E. coli*) DH5α cells (Shenggong Corporation, Shanghai, China) containing pcDNA3-X were cultured overnight on an LB plate containing 50 *μ*g/ml ampicillin. Single colonies were selected and inoculated into 3 ml of LB broth containing 50 *μ*g/ml ampicillin and incubated for 8 h at 37°C; subsequently, the broth was examined for cloudiness, which signifies bacterial growth. The preparation and purification of pcDNA3-X were carried out according to the manufacturer’s instructions using the Plasmid DNA purification system (Promega Biosciences LLC).

HBx primers were designed using sequences obtained from the National Center for Biotechnology Information (NCBI) containing the *Eco*RI and *Pst*I sites. The sequences of the primers with the restriction enzyme sites underlined are as follows: P1, 5′-CGGAATTCGCCACCATGGCTGCTAGGCTGTGC-3′; P2, 5′-CCCCTGCAGTTAGCGAAGTGCACACGGTCC-3′; P3, 5′-CCCCTGCAGTTACCAGTCTTTAAACAAACAGTC-3′; and P4, 5′-CCCCTGCAGAGATGATTAGGCAGAGGTGA-3′.

The primer pairs, P1/P2, P1/P3 and P1/P4, were used to amplify HBx X1, HBx X2 and the full-length HBx gene, respectively. The thermocycling conditions for polymerase chain reaction (PCR) were as follows: initial denaturation at 94°C for 5 min; 30 cycles of denaturation at 94°C for 45 sec, annealing at 55°C for 45 sec and extension at 72°C for 1 min; followed by a final extension at 72°C for 10 min. The PCR products were separated by electrophoresis on a 1.3% agarose gel. Subsequently, 216-bp (X1) and 352-bp (X2) fragments were collected and purified using the DNA gel extraction kit following the manufacturer’s instructions (Shanghai Sangon Biotech).

The pAS2-1 plasmid was extracted from *E. coli* DH5α cells using the Plasmid DNA purification system, following the manufacturer’s instructions (Promega Biosciences LLC). Following double digestion with *Eco*RI and *Pst*I, the pAS2-1 fragments were separated by electrophoresis in a 0.6% agarose gel. Fragments with a size of 8,400 bp were collected and purified using the DNA gel extraction kit.

The extracted 8400-bp fragment was ligated with the X1 or X2 fragments using T4 DNA ligase overnight at 4°C. The recombinant plasmids were transfected into the *E. coli* DH5α cells and cultured overnight at 37°C before plating on LB medium containing 50 *μ*g/ml ampicillin and were again cultured overnight at 37°C. Colonies on the LB plate were selected and inoculated into 3 ml of LB broth containing 50 *μ*g/ml ampicillin, incubated for 8 h at 37°C and examined for cloudiness in the broth, which signifies bacterial growth. The plasmids were extracted, purified and sent to Life Technologies Corp., (Shanghai, China), for sequencing. The recombinant plasmids were designated as pAS2-1-X1 and pAS2-1-X2. The X mutant fragments were also cloned into the pUCm-T vector, creating pUCm-T-X1 and pUCm-T-X2. The X mutant fragments were excised from pUCm-T-X1 and pUCm-T-X2 using the *Eco*RI and *Xho*I restriction enzymes and ligated into the pCMV-HA vector that had been linearized with the same enzymes.

### Plasmid transfection and translation of fusion proteins in yeast cells

The screening procedure used in this study was a modification of the method previously described by Gietz *et al* (7). The lithium acetate method (7) was used to transfect *Saccharomyces cerevisiae* AH109 with the pAS2-1 plasmids (pAS2-1-X1, pAS2-1-X2, pAS2-1-X and pAS2-1), as well as pCL1 and pLAM5′-1 plasmids into the cells. Transfectants were grown either in YPD medium (10 g/l yeast extract, 20 g/l peptone and 20 g/l dextrose) or synthetic minimal medium (0.67% yeast nitrogen base, 2% dextrose and appropriate auxotrophic supplements) at 30°C. The plasmids, pCL1 and pLAM5′-1, were included as positive and negative controls, respectively. The transfectants were plated on *SC/-trp-leu-his* medium selective for histidine, leucine and tryptophan prototrophy. The AH109 transfectants were designated as AH109-pAS2-1-X1, AH109-pAS2-1-X2, AH109-pAS2-1-X, AH109-pAS2-1, AH109-pLAM5′-1 and AH109-pCL1. The X1 genes in AH109-pAS2-1-X1 and AH109-pAS2-1-X2 were detected by PCR. The X1 and X2 binding domain (X1-BD and X2-BD) fusion proteins were detected in the AH109 strain by western blot analysis. Cell lysates were blocked with non-fat dried milk and exposed to a 1:3,000 dilution of binding domain (BD) monoclonal antibody at 4°C overnight. After washing with Tris-buffered saline/Tween-20, the secondary antibody, alkaline phosphatase-conjugated goat anti-rabbit IgG, was added followed by incubation for 2 h at room temperature. Proteins were visualized with 5-bromo-4-chloro-3-indolyl phosphate and nitro blue tetrazolium. In addition, freshly grown yeast transformant colonies were assayed for β-galactosidase (β-gal) activity by replica plating onto Whatman filter papers that were snap-frozen twice for 10 sec in liquid nitrogen and incubated at 30°C for 8 h in a buffer containing 5-bromo-4-chloro-3-indolyl-β-D-galactopyranoside solution. Positive interactions were detected by the appearance of blue colonies.

### Mating assays

Y187-pACT2-COXIII single colonies were streaked as parallel lines on *SC/-leu* medium at 30°C for 2 days, as were the AH109-pAS2-1-X1, AH109-pAS2-1-X2, AH109-pAS2-1-X, AH109-pAS2-1, AH109-pLAM5′-1 and AH109-pCL1 colonies on *SC/-trp* medium. The two types of transformants were then mated and incubated in YPD medium at 30°C for 4 h. Finally, the transformants were subcultured on *SC/-leu-trp* medium, incubated at 30°C for 2 days, and assayed for *LacZ* activity. Freshly grown colonies on *SC/-leu-trp* medium were selected and subcultured on the same medium. β-gal activity was assayed by replica plating of the yeast transformants onto Whatman filter papers. The filters were snap-frozen twice for 10 sec in liquid nitrogen and incubated at 30°C for 8 h in buffer containing 5-bromo-4-chloro-3-indolyl-β-D-galactopyranoside solution. Positive interactions were detected by the appearance of blue colonies.

### Transient transfection, co-immunoprecipitation and western blot analysis

The transient transfection of COS7 cells (Typical Culture Preservation Commission Cell Bank, Chinese Academy of Sciences, Shanghai, China) was used to detect the ectopic expression of the HBV X1, HBV X2 and COXIII proteins. The COS7 cells were grown in DMEM (90% Dulbecco’s modified Eagle’s medium, 10% fetal bovine serum); 5×10^6^ COS7 cells were transfected with 10 *μ*g of plasmid DNA using the liposome TransFast transfection reagent kit (Promega Biosciences LLC). Forty-eight hours after transfection, the cells were washed 3 times with ice-cold phosphate-buffered saline (PBS) and lysed with lysis buffer supplemented with the protease inhibitors provided with the Protein G Immunoprecipitation kit (Promega Biosciences LLC). Appropriate protein expression was verified by gel electrophoresis of cellular lysates and immunoblotting with specific antibodies. To prevent N-linked glycosylation during protein synthesis, the cells were treated with tunicamycin for 2 h prior to transfection and the tunicamycin level was maintained during the transient transfection period. c-Myc monoclonal antibody was used to recognize the expression of the tagged Myc-COXIII protein. For co-immunoprecipitation, 1 ml of cell lysate was incubated for 3 h at 4°C with 50 *μ*l of a 50% suspension of protein G agarose that had been pre-coated with 3 *μ*g of c-Myc antibody. The immunocomplex was washed 3 times with ice-cold lysis buffer and resuspended in sodium dodecyl-sulfate polyacrylamide gel electrophoresis (SDS-PAGE) sample buffer; electrophoresis was performed, and the proteins were electroblotted onto a nitrocellulose membrane. The blotting membrane was blocked with non-fat dried milk and exposed to a 1:200 dilution of HA polyclonal antibody for 1 h. After washing with Tris-buffered saline Tween-20, the proteins on the nitrocellulose membrane were detected using an electrochemiluminescence kit (Boster Biotech) that included a goat anti-rabbit antibody, following the manufacturer’s instructions. The COS7 cells transfected with the pCMV-Myc vector or with the pCMV-HA-X vector and non-transfected cells were used as negative controls; COS7 cells transfected with the pCMV-Myc-COXIII or pCMV-HA-X vector were used as positive controls.

## Results

### Plasmid construction

**Figure 1:**
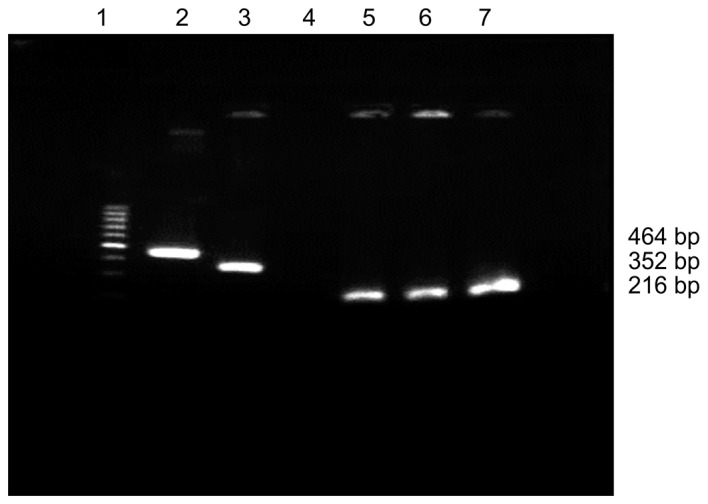
Hepatitis B viral virus X protein (HBx) fragments amplified by PCR from pAS2-1 recombinant plasmid and separated by gel electrophoresis (0.6% agarose). The different fragments have the expected molecular weights. Lane 1, 100-bp DNA ladder used as molecular weight marker; lane 2, full length (464 bp) HBx gene; lane 3, HBx X2 (352 bp) fragment; lane 4, negative controls; lanes 5–7, HBx X1 (216 bp) fragment.

The HBx X1 and X2 amplified fragments were ligated with pAS2-1 following a double restriction enzyme digestion. The recombinant plasmids were designated as pAS2-1-X1 and pAS2-1-X2. The PCR-amplified fragments from pAS2-1-X1, pAS2-1-X2 and pAS2-1-X were separated electrophoretically on a 0.6% agarose gel (Fig. 1).

### Sequencing of HBx X1 in pAS2-1-X1 and HBx X2 in pAS2-1-X2

**Figure 2:**
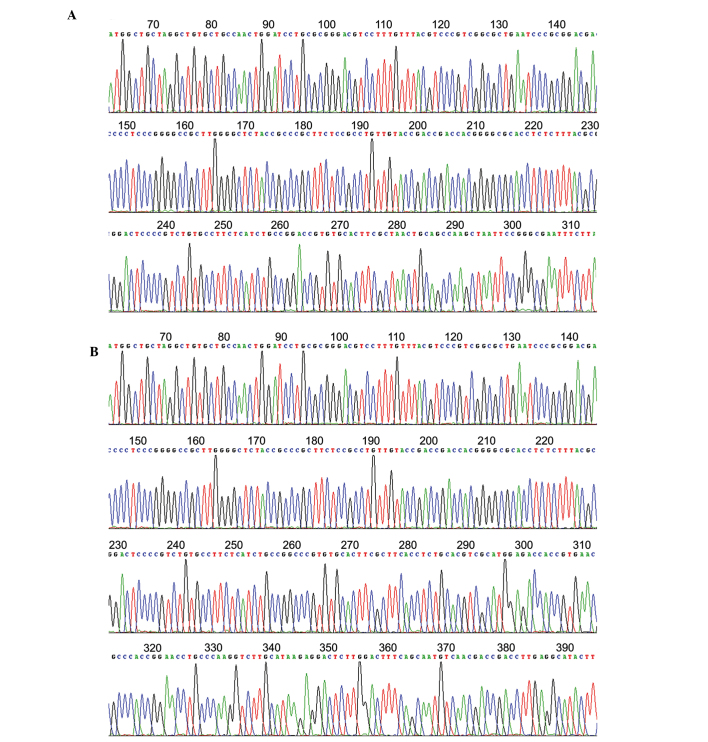
(A) DNA sequence of hepatitis B viral virus X protein (HBx) X1. Amplified HBx X1 fragments were ligated with linearized pAS2-1 and the recombinant plasmid designated pAS2-1-X1. Purified pAS2-1-X1 was sent to Life Technologies Corp. for sequencing. The X1 gene has 98% homology with the X gene from the GenBank database. The X1 gene runs from base 63 to base 278. (B) DNA sequence of HBx X2. Amplified HBx X2 fragments were ligated with linearized pAS2-1 and the recombinant plasmid designated pAS2-1-X2. The sequence shows that the X2 gene has 98% homology with X gene from the GenBank database. The X2 gene runs from base 61 to base 412.

The amplified HBx X1 and X2 fragments were ligated with pAS2-1 after a double restriction enzyme digestion. The recombined plasmids were designated as pAS2-1-X1 and pAS2-1-X2. Purified recombined plasmids were sent to Life Technologies Corp., Shanghai Branch for sequencing. The HBx X1 and X2 genes were 98% homologous with the X gene from GenBank (Fig. 2).

### Expression of fusion proteins in yeast cells

**Figure 3:**
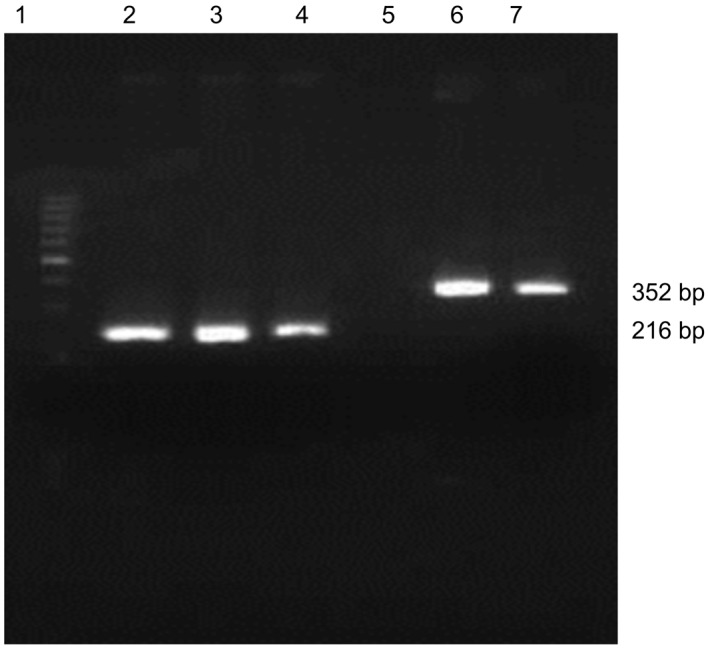
Hepatitis B viral virus X protein (HBx) X1 and X2 fragments amplified by PCR from AH109-pAS2-1-X1 and AH109-pAS2-1-X2. X1 and X2 fragments of the expected size can be seen after gel electrophoresis (0.6% agarose). Lane 1, 100-bp DNA ladder; lanes 2–4, HBx X1 (216 bp) amplified with primers P1 and P2; lane 5, negative controls; lanes 6 and 7, HBx X2 (352 bp) amplified with primers P1 and P3.

**Figure 4:**
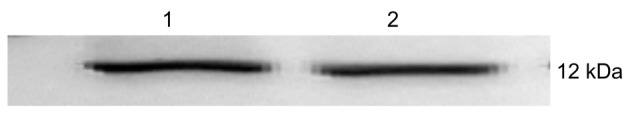
X1- and X2-binding domain (X1-BD and X2-BD) fusion proteins expressed in the yeast AH109 and detected by western blot analysis. The molecular weights of the 2 fusion proteins are approximately 9 and 12 kDa. Lane 1, X1-BD; lane 2, X2-BD.

The pAS2-1-X1 and pAS2-1-X2 recombinant plasmids were transfected into the AH109 yeast cells, creating AH109-pAS2-1-X1 and AH109-pAS2-1-X2, respectively. The presence of HBx X1 and X2 was detected by PCR (Fig. 3). The expression of the two fusion proteins and the X1 and X2 binding domains (X1-BD and X2-BD) in the AH109 strain was detected by western blot analysis (Fig. 4).

### Detection of β-gal activity

**Figure 5:**
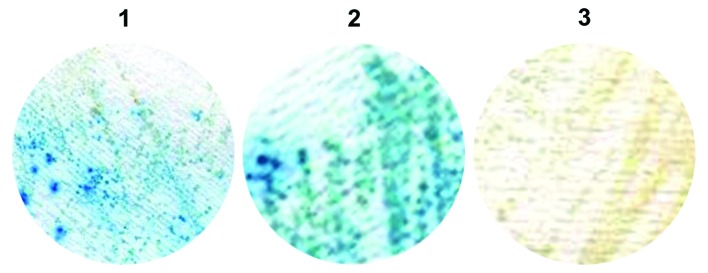
Assays to detect β-galactosidase (β-gal) activity after mating. Panel 1, blue colonies on Whatman filter paper after mating between Y187-pACT2-COXIII and AH109-pAS2-1-X; panel 2, blue colonies on Whatman filter paper after mating between Y187-pACT2-COXIII and AH109-pAS2-1-X2; panel 3, white colonies on Whatman filter paper after mating between Y187-pACT2-COXIII and AH109-pAS2-1-X1.

Following the elimination of the auto-activation of X1-BD and X2-BD in AH109, freshly grown clones on an *SC/-leu-trp* medium were selected and subcultured on the same medium. β-gal activity was assayed by replica plating of the yeast transformants onto Whatman filter papers. Positive interactions were detected by the appearance of blue colonies (Fig. 5).

### Co-immunoprecipitation and western blot analysis for detecting interactions between HBx X1 or HBx X2 proteins with COXIII in COS7 cells

**Figure 6:**
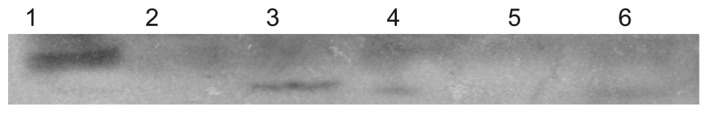
Co-immunoprecipitation and western blot anlaysis were performed to detect interactions between X or X mutant proteins with cytochrome c oxidase subunit III (COXIII) in COS7 cells. Lane 1, western blot analysis using hemagglutinin (HA) antibody shows that a protein of approximately 17 kDa (the predicted size of HA-X) was co-immunoprecipitated with v-myc myelocytomatosis viral oncogene homologue (Myc) antibody from an extract of COS7 cells containing HA-X and Myc-COXIII fusion proteins; lane 2, western blot analysis of an extract from COS7 cells transformed with pCMV-HA-X 1 and pCMV-Myc-COXIII plasmids; lane 3, western blot analysis using HA antibody showing a protein of approximately 14 kDa (the predicted size of HA-X2) was co-immunoprecipitated with Myc antibody from an extract of COS7 cells containing HA-X2; lane 4, western blot analysis of an extract from COS7 cells transformed with plasmids pCMV-HA-X and pCMV-Myc; lane 5, western blot analysis of an extract from COS7 cells transformed with plasmids pCMV-HA and pCMV-Myc; lane 6, western blot analysis of an extract from non-transfected COS7 cells.

COS7 cells co-transformed using the plasmids pCMV-HA-X, pCMV-HA-X1, pCMV-HA-X2 or pCMV-Myc-COXIII or with the pCMV-HA-X and pCMV-Myc plasmids, were extracted and immunoprecipitated with Myc antibody. A protein of approximately 17 kDa (the predicted size of HA-X) was co-immunoprecipitated with Myc antibody from cells containing HA-X and Myc-COXIII fusion protein, and a protein of approximately 14 kDa (the predicted size of HA-X2) was co-immunoprecipitated with Myc antibody from cells containing HA-X2 and Myc-COXIII fusion protein, but no co-immunoprecipitation was observed from the cells co-transformed with pCMV-HA-X1 and pCMV-Myc-COXIII or with pCMV-HA-X and pCMV-Myc. The HA-X and HA-X 2 proteins were recognized by immunoblotting with HA antibody. These results confirm that aa72-117 of HBx include the key peptide(s) for the combination of HBx and COXIII (Fig. 6).

## Discussion

The HBV genome contains four functionally distinct open reading frames on one strand in the same transcriptional polarity. The X gene is the smallest, coding for a regulatory protein of 154 aa, and is known to exhibit a transcriptional transactivation function through the X-responsive elements of many viral and cellular genes implicated in the carcinogenicity of this virus (8,9). One of the basic properties of HBx is that it has no DNA binding activity; therefore, its transactivation and/or oncogenic function is possibly exerted through interaction(s) with cellular protein(s) (2). HBx is predominantly localized in the cytoplasm and its presence there is required for it to function (2,10); therefore, the direct cellular target of HBx may also be located in the cytoplasm. The identification of this target represents a major goal in defining the functions of HBx in HBV replication and HCC. In our previous study (6), we detected and cloned a novel HBx-interactive protein from a normal human liver cDNA library. Sequence analysis revealed that it was a homolog of *Homo sapiens* COXIII. In addition, using confocal microscopy, our laboratory has recently demonstrated that HBx co-localizes with inner mitochondrial protein COXIII in HL7702 cells (11).

There is considerable evidence that HBx is involved in the dysfunction of cytochrome *c* oxidase (10,12), the terminal enzyme of the mitochondrial respiratory chain that catalyzes the transfer of electrons from reduced cytochrome *c* to molecular oxygen. COXIII, which is encoded by mitochondrial DNA and is synthesized within the mitochondria, is a large COX subunit and acts as the catalytic core of the enzyme (13). COXIII is a 7 transmembrane helix protein and binds to subunit I. The N-terminal region of subunit III is adjacent to D132 of subunit I, the initial proton acceptor of the D pathway that transfers protons from the protein surface to the buried active site located 30 Å away (14). One of the roles of subunit III in the normal oxidase is to maintain rapid proton uptake through the D pathway at a physiological pH (14). Single-turnover experiments have indicated that proton uptake through the D pathway at pH 8.0 is reduced from >10,000 s^−1^ in the presence of subunit III to 350 s^−1^ in its absence. Part of a proton antenna for the D pathway may be lost upon the removal of subunit III (15). Based on these facts, when the proton-transferring function of COXIII is inhibited, the electrochemical gradient across the inner mitochondrial membrane decreases and triggers the opening of the mitochondrial permeability transition pore (MPTP), which results in the elevation of cytosolic calcium levels. This causes calcium overload, leading to mitochondrial disruption and hepatic cell injury (16,17). Since there is a long time gap (chronic infection) between HBV infection and liver carcinogenesis, the chronic hepatic cell injury caused by HBx should be focused on to a greater extent in future studies.

Identification of the precise location for the interaction between HBx and COXIII is crucial for clarifying the precise mechanisms involved in HBx co-localization with the mitochondria. A genetic approach, the yeast two-hybrid system, is a powerful method for mapping the essential binding sites of proteins of interest. In the present study, we successfully constructed two plasmids that produce mutants of HBx X1 (aa1-72) or HBx X2 (aa1-117). The interaction of HBx with COXIII was identified using the yeast two-hybrid system. That this binding is specific for HBx as supported by the histidine-independent growth and blue-colony formation in the β-gal assay by yeast cells harboring both pAS-2-1-X2 and pACT2-COXIII recombinant plasmids, as well as by the behavior of the cells in false-positive elimination tests. Furthermore, the outcome of co-immunoprecipitation experiments confirmed the specificity of the interaction between HBx X2 mutant proteins and COXIII. From these data, it is reasonable to conclude that aa72-117 of HBx encode the peptides essential for HBx to bind with COXIII.

Among mammalian hepadnavirus genomes, there are many conserved HBx regions, and these seem to play an important role in HBx function. For example, Misra *et al* (18) demonstrated that the conserved amino-terminal region (aa1-20) of HBx has a transrepression function, and Kumar *et al* (19) demonstrated that a truncated mutant (residues, aa 58-140) of HBx effecientlyh stimulates the Rous sarcoma virus long terminal repeat (RSV-LTR). It has also been demonstrated that aa68-117 in HBx are sufficient for HBx localization in the mitochondria and cell death induction, which is consistent with our conclusions, and this effect is independent of its transactivation activity (12). In studies investigating HBx function, it is important to map the site of its interaction with the mitochondria. In the present study, our findings support the view that aa72-117 of HBx form the key domain for the combination of HBV with COXIII, which may play a crucial role in the function of HBV in the mitochondria.

In conclusion, our data led us to the hypothesis that HBx may impair the mitochondrial respiration chain and energy metabolism through the association between HBx and COXIII. Future investigation are required to focus on the physiological significance of HBx interactions with COXIII and make the peptides in aa72-117 of HBx a novel target for further research on and in the treatment of tumorigenesis in HCC.
